# Age-Associated Seroprevalence of Coronavirus Antibodies: Population-Based Serosurveys in 2013 and 2020, British Columbia, Canada

**DOI:** 10.3389/fimmu.2022.836449

**Published:** 2022-03-23

**Authors:** Guadalein Tanunliong, Aaron C. Liu, Samantha Kaweski, Mike Irvine, Romina C. Reyes, Dale Purych, Mel Krajden, Muhammad Morshed, Inna Sekirov, Soren Gantt, Danuta M. Skowronski, Agatha N. Jassem

**Affiliations:** ^1^ Department of Pathology and Laboratory Medicine, University of British Columbia, Vancouver, BC, Canada; ^2^ Department of Experimental Medicine, University of British Columbia, Vancouver, BC, Canada; ^3^ British Columbia Centre for Disease Control Public Health Laboratory, Vancouver, BC, Canada; ^4^ British Columbia Centre for Disease Control, Vancouver, BC, Canada; ^5^ Faculty of Health Sciences, Simon Fraser University, Burnaby, BC, Canada; ^6^ LifeLabs, Burnaby, BC, Canada; ^7^ Surrey Memorial Hospital, Fraser Health Authority, Surrey, BC, Canada; ^8^ Departments of Pediatrics and Microbiology, Infectious Diseases & Immunology, University of Montreal, Montreal, QC, Canada; ^9^ Sainte-Justine University Hospital Centre, Montreal, QC, Canada; ^10^ School of Population and Public Health, University of British Columbia, Vancouver, BC, Canada; ^11^ Communicable Diseases and Immunization Services, British Columbia Centre for Disease Control, Vancouver, BC, Canada

**Keywords:** SARS-CoV-2, COVID-19, antibodies, humoral immune response, endemic coronavirus, cross-reactive antibodies, COVID-19 severity

## Abstract

**Background:**

Older adults have been disproportionately affected during the SARS-CoV-2 pandemic, including higher risk of severe disease and long-COVID. Prior exposure to endemic human coronaviruses (HCoV) may modulate the response to SARS-CoV-2 infection and contribute to age-related observations. We hypothesized that cross-reactive antibodies to SARS-CoV-2 are associated with antibodies to HCoV and that both increase with age.

**Methods:**

To assess SARS-CoV-2 unexposed individuals, we drew upon archived anonymized residual sero-surveys conducted in British Columbia (BC), Canada, including before SARS-CoV-2 emergence (May, 2013) and before widespread community circulation in BC (May, 2020). Fifty sera, sex-balanced per ten-year age band, were sought among individuals ≤10 to ≥80 years old, supplemented as indicated by sera from March and September 2020. Sera were tested on the Meso Scale Diagnostics (MSD) electrochemiluminescent multiplex immunoassay to quantify IgG antibody against the Spike proteins of HCoV, including alpha (HCoV-229E, HCoV-NL63) and beta (HCoV-HKU1, HCoV-OC43) viruses, and the 2003 epidemic beta coronavirus, SARS-CoV-1. Cross-reactive antibodies to Spike, Nucleocapsid, and the Receptor Binding Domain (RBD) of SARS-CoV-2 were similarly measured, with SARS-CoV-2 sero-positivity overall defined by positivity on ≥2 targets.

**Results:**

Samples included 407 sera from 2013, of which 17 were children ≤10 years. The 2020 samples included 488 sera, of which 88 were children ≤10 years. Anti-Spike antibodies to all four endemic HCoV were acquired by 10 years of age. There were 20/407 (5%) sera in 2013 and 8/488 (2%) in 2020 that were considered sero-positive for SARS-CoV-2 based on MSD testing. Of note, antibody to the single SARS-CoV-2 RBD target was detected in 329/407 (81%) of 2013 sera and 91/488 (19%) of 2020 sera. Among the SARS-CoV-2 overall sero-negative population, age was correlated with anti-HCoV antibody levels and these, notably 229E and HKU1, were correlated with cross-reactive anti-SARS-CoV-2 RBD titres. SARS-CoV-2 overall sero-positive individuals showed higher titres to HCoV more generally.

**Conclusion:**

Most people have an HCoV priming exposure by 10 years of age and IgG levels are stable thereafter. Anti-HCoV antibodies can cross-react with SARS-CoV-2 epitopes. These immunological interactions warrant further investigation with respect to their implications for COVID-19 clinical outcomes.

## Introduction

Severe Acute Respiratory Syndrome Coronavirus 2 (SARS-CoV-2) is the causative agent of the Coronavirus Disease 2019 (COVID-19) pandemic, responsible for hundreds of millions of known infections worldwide since its emergence in December 2019 (1). While younger children more often experience mild or asymptomatic infections, older adults are disproportionately affected by COVID-19 (2, 3), with a strong association between morbidity and mortality (4). Older adults also have an increased risk of prolonged symptoms following COVID-19, or “long COVID syndrome”. In contrast, COVID-19 rarely causes serious disease in children, emphasizing the urgent need to better understand the pathogenesis of age-dependent disease. Several hypotheses have been proposed to describe the age-related differences in COVID-19 severity (3), including comorbidities, immune senescence, differential expression of the SARS-CoV-2 host receptor angiotensin-converting enzyme 2 (5), as well as variation in the inflammatory immune response; however, none appear to account for the strong correlation between COVID-19 severity and age. Additionally, immunological interactions due to prior exposure to closely-related pathogens may also modulate the immune response to SARS-CoV-2, which may potentially enhance or dampen protective immune responses and contribute to COVID-19 severity.

In addition to the betacoronavirus SARS-CoV-2, humans are also susceptible to at least six other viruses within the alphacoronavirus and betacoronavirus genus of the *Coronaviridae* family (6). Among these, SARS-CoV-1 and Middle East Respiratory Syndrome (MERS-CoV) are two highly pathogenic betacoronaviruses, which are associated with elevated morbidity and mortality rates among those infected (7). In contrast, there are four endemic human coronaviruses (HCoVs), two alphacoronaviruses (HCoV-NL63 and HCoV-229E) and two betacoronaviruses (HCoV-HKU1 and HCoV-OC43), which circulate seasonally and typically only cause mild upper respiratory tract infections (8–10). Initial infection generally occurs during childhood, with virtually everyone believed to have some immunity against HCoVs by adolescence (9, 11). However, HCoV immunity wanes (12), hence reinfections are common, typically occurring every 2-3 years throughout a person’s lifetime (6, 13).

Observational studies have identified the presence of SARS-CoV-2 cross-reactive immune responses in pre-pandemic individuals unexposed to SARS-CoV-2, including cross-reactive T cells (14), B cells (15, 16), and antibody responses (17–20). To date, the source of these cross-reactive immune responses, as well as their potential negative or positive implications during a SARS-CoV-2 infection are still poorly understood. As HCoVs are highly similar to SARS-CoV-2 (6), it has been speculated that the cross-reactive SARS-CoV-2 antibody responses seen in unexposed individuals might be associated with pre-existing HCoV antibodies.

We hypothesized that cross-reactive antibodies against SARS-CoV-2 are associated with the presence of HCoV antibodies in SARS-CoV-2-unexposed individuals and that both increase with age. To assess the age-associated seroprevalence of HCoV and SARS-CoV-2 cross-reactive antibodies among SARS-CoV-2 unexposed individuals, we drew upon archived samples of anonymized residual sero-surveys previously conducted according to a cross-sectional, age-stratified protocol in British Columbia, Canada before SARS-CoV-2 emergence (May, 2013) or its broad pandemic circulation (May, 2020).

## Materials and Methods

### Study Population

As part of risk assessment for emerging respiratory pathogens, the British Columbia Centre for Disease Control (BCCDC) previously established a sero-survey protocol to assess changes in sero-positivity with time (21). As part of this protocol, anonymized residual sera are periodically procured in an age-based fashion from the only outpatient laboratory network (formerly BC Biomedical Laboratories, now LifeLabs) servicing the Lower Mainland, BC (i.e. Greater Vancouver Area, including the Fraser Valley), where ~60% of the provincial population resides and community attack rates are expected to be high (21). For the current study, remaining archived samples primarily collected during prior sero-surveys for influenza (May, 2013) and SARS-CoV-2 (May, 2020) were used (21).

For this study, remaining archived specimens were included in the study if at least 200µL were available. Accompanying characteristics included age and sex. The original May 2013 sero-survey protocol sought up to 50 samples per ten-year age band <10, 10-19, 20-29, 30-39, 40-49, 50-59, 60-69, 70-79, ≥ 80 years of age; whereas, the May 2020 protocol sought 100 serum samples among those <5 and 5-9 years and per ten-year age band thereafter. When available, up to 50 sera per age-band and season were randomly selected (sex-balanced) where more than 50 were an option. Where <50 sera per age band were available, notably among children, additional samples were supplemented from a similarly conducted sero-survey in March 2020. As the samples from 2013 were collected prior to the COVID-19 pandemic, they were assumed to be from persons unexposed to SARS-CoV-2 and all deemed “true SARS-CoV-2 sero-negatives”. Of note, SARS-CoV-2 circulation was well-suppressed in BC through spring 2020 with sero-surveys in both March and May 2020 showing overall <1% sero-positivity at most (21).

We further assessed 40 anonymized residual samples identified from the serial SARS-CoV-2 sero-surveys, similarly-conducted March (N=11), May (N=9) and September 2020 (N=20) sero-surveys, respectively. All 40 samples were known to be positive for antibodies to at least one of three SARS-CoV-2 antigens (Spike, S1 Receptor Binding Domain (RBD), Nucleocapsid) using commercial Health Canada-approved chemiluminescent immuno-assays (CLIA) (21). Among these, 20/40 had been identified as positive for at least two CLIA targets (2, 4 and 14 from March, May and September, respectively), while the remaining 20/40 were positive only for one CLIA target. Among these, the 19 that were found to be SARS-CoV-2 sero-positive according to the Meso Scale Discovery (MSD) testing algorithm (described below) are included as a subset of positive controls for comparison, when appropriate.

Original sero-surveys and use of specimens for these investigations were approved by the Clinical Research Ethics Board of the University of British Columbia (H20-00653).

### Meso Scale Discovery (MSD) Multiplexed Pan-Coronavirus Immunoassay

To simultaneously detect and quantify antibody levels against six coronaviruses (all except MERS), we utilized a highly sensitive multiplexed chemiluminescent immunoassay from MSD (V-PLEX Coronavirus Panel 2). Multi-spot plates spotted with purified antigens were used for the detection of IgG antibodies against Spike, S1 RBD, and Nucleocapsid of SARS-CoV-2, as well as Spike of SARS-CoV-1 and alpha-HCoVs (229E and NL63) and beta-HCoVs (HKU1 and OC43). Assays were performed according to the manufacturer’s protocol.

Briefly, multi-spot plates were initially incubated with MSD Blocker A for 30 minutes, then washed off. Reference standard, controls, and serum samples (diluted 1:5000 in Diluent 100) were added and incubated on the plates for 2 hours. Plates were washed and MSD SULFO-TAG Anti-Human IgG detection antibody was added, incubated for an hour, and then washed again. Finally, MSD Gold Read Buffer B was added to the plate and signals were immediately measured on the MSD QuickPlex SQ120 instrument. All incubation steps were carried out at room temperature while shaking at 700rpm, and all wash steps were performed three times with MSD Wash Buffer, prior to addition of the subsequent reagents.

Raw data generated was processed using MSD Discovery Workbench software (Version 4.0), then imported into RStudio (Version 1.2.5033) to interpret signal cut-off values. SARS-CoV-2 cut-off thresholds for reactivity provided by the manufacturer are as follows: anti-SARS-CoV-2 Spike values above 1960 AU/mL, anti-SARS-CoV-2 Nucleocapsid values above 5000 AU/mL, and anti-SARS-CoV-2 S1 RBD values above 538 AU/mL. Samples above cut-off values for at least two of three SARS-CoV-2 targets (S1 RBD and/or nucleocapsid, and/or spike) were considered serologically reactive using this MSD immunoassay (i.e. overall status sero-positive) based on our previous validation (22).

Because everyone is expected to have been exposed to HCoV by adolescence, we opted to use the lower 95% confidence interval of the geometric mean antibody titres among the SARS-CoV-2 negative population (which includes all of the 2013 population and the SARS-CoV-2 sero-negatives from 2020) to define positive signal cut-offs for HCoV. Consequently, the positivity cutoffs assigned for HCoVs were as follows: 1700 AU/mL for HCoV-229E Spike, 900 AU/mL for HCoV-HKU1, 270 AU/mL for HCoV-NL63, and 2000 AU/mL for HCoV-OC43. As only 1 epitope (Spike) was included for HCoVs, sero-positivity for HCoVs was defined based on positivity for the Spike epitope alone.

### Statistical Analysis

All statistical analyses were conducted on R (Version 3.6.2) and RStudio (Version 1.2.5033). Processed data from the MSD immunoassay was visualized using the *ggplot2* (Version 3.3.3) and *ggpubr* (Version 0.6.0) packages on RStudio. Kruskal-Wallis, Wilcoxon rank sum, Spearman’s correlation calculations were conducted using both *ggpubr* and *stats* (Version 3.6.2) packages. Correlation matrices were made using *corrplot* (Version 0.92). Geometric mean antibody levels were calculated on *rcompanion* (Version 2.4.1). Antibody trends over chronological age were fitted using a locally weighted regression to fit a curve between points on *ggpubr*. Using the *stats* package, multivariable linear regression models were built, model diagnostics and model fit were assessed, and distribution of residuals were evaluated to determine whether assumptions for linear models were met. Box-Cox transformations on *MASS* (Version 7.3-54) were used to determine the optimal transformation methods. All regression analyses were carried out using natural log-transformed antibody levels. P-values less than 0.05 were considered statistically significant.

## Results

### Study Population

A total of 935 sera were assessed from individuals ranging 0-99 years of age. A summary of the age and sex distribution of individuals is listed in **Table 1**, according to the 10-year age bands from our sampling. Of the 935 sera, 895 were used for sero-prevalence estimation and 40 were previously identified as sero-positive for SARS-CoV-2 on at least one commercial CLIA. There were 407 samples procured from 2013 and 528 from 2020. Excluding the 40 sera from 2020 that were sero-positive for SARS-CoV-2 on commercial platforms, 488 samples from the 2020 season were used including 463 from May 2020, and 25 from March 2020. Overall, the 2013 population tested was 62.4% (254/407) female, while the 2020 population (March and May taken together) was 50.8% (248/488) female.

**Table 1:** Age and sex distribution of study participants from 2013 and 2020.

	2013 (N = 407)		2020 (N = 488)		Overall (N = 895)	
	F (N = 254)	M (N = 153)	F (N= 248)	M (N = 240)	2013	2020
Median Age (IQR)	42 (28, 66)	58 (30, 73)	40 (17, 64)	40 (19, 65)	49 (28, 69)	40 (18, 65)
**Age Band**						
**0-1**	0 (0%)	0 (0%)	1 (0.4%)	3 (1.2%)	0 (0%)	4 (0.8%)
**2-4**	5 (2%)	2 (1.3%)	22 (8.9%)	12 (5.0%)	7 (1.7%)	34 (7%)
**5-9**	4 (1.6%)	6 (3.9%)	25 (10%)	25 (10%)	10 (2.5%)	50 (10%)
**10-19**	32 (13%)	16 (10%)	25 (10%)	25 (10%)	48 (12%)	50 (10%)
**20-29**	34 (13%)	14 (9%)	25 (10%)	25 (10%)	48 (12%)	50 (10%)
**30-39**	45 (18%)	4 (2.6%)	25 (10%)	25 (10%)	49 (12%)	50 (10%)
**40-49**	30 (12%)	19 (12%)	25 (10%)	25 (10%)	49 (12%)	50 (10%)
**50-59**	25 (9.8%)	24 (16%)	25 (10%)	25 (10%)	49 (12%)	50 (10%)
**60-69**	28 (11%)	20 (13%)	25 (10%)	25 (10%)	48 (12%)	50 (10%)
**70-79**	21 (8.3%)	29 (19%)	25 (10%)	25 (10%)	50 (12%)	50 (10%)
**80+**	30 (12%)	19 (12.4%)	25 (10%)	25 (10%)	49 (12%)	50 (10%)

Of the 40 sera previously identified as sero-positive on at least one CLIA platform, we identified 19/40 as sero-positive by a validated MSD algorithm (22), which requires a positive result on 2 of the 3 SARS-CoV-2 targets tested. 1/19 was previously positive for only one CLIA target and was a sample collected in March 2020, and the remaining 18/19 were previously positive for at least two CLIA targets (4 were collected in May and 14 were collected in September 2020). We included these 19 MSD-positive specimens as our SARS-CoV-2 sero-positive controls.

### HCoV Antibodies Across Age, and by Sex and Year

**Figure 1:**
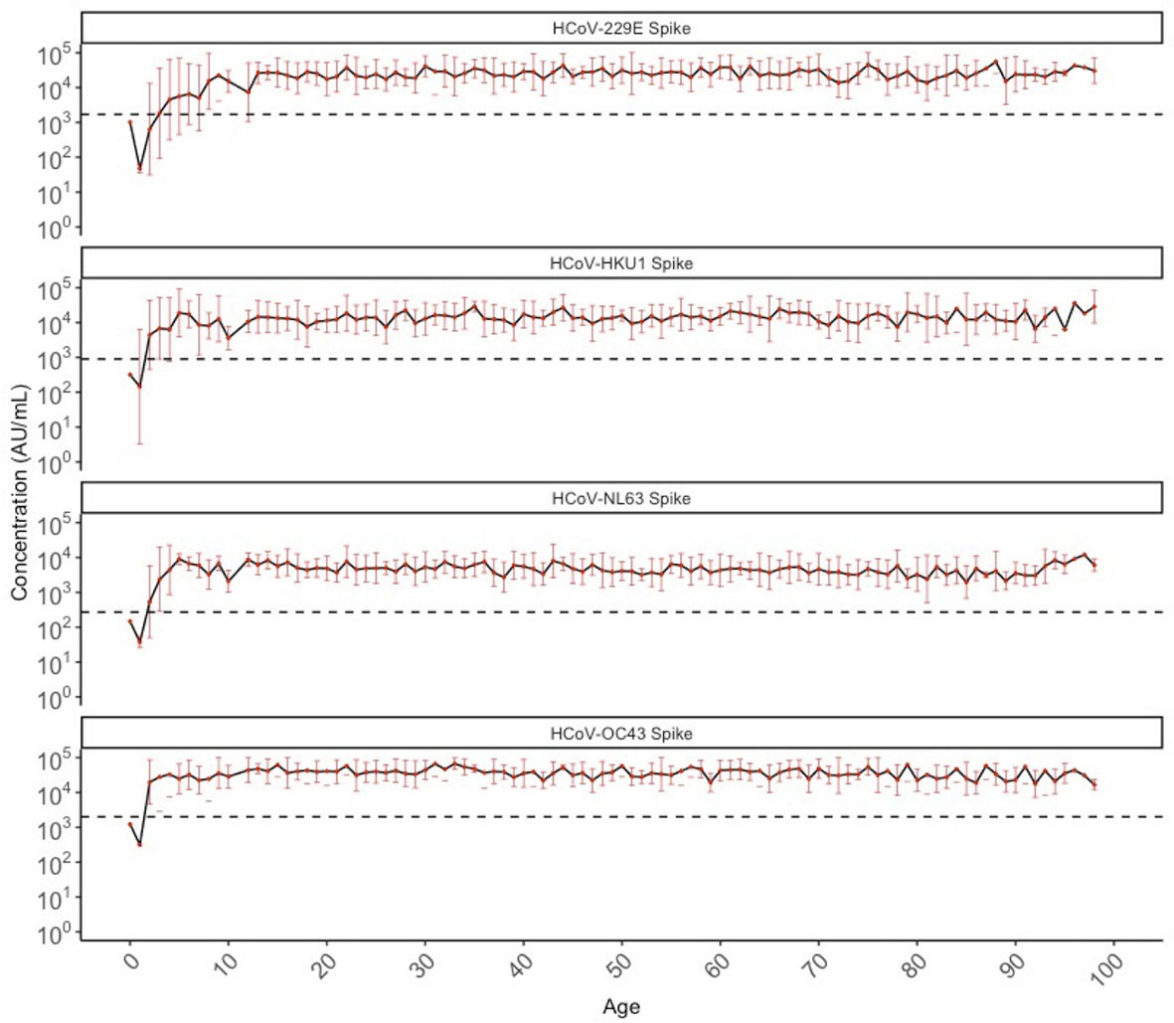
Geometric mean IgG antibody titres against HCoV-229E, HCoV-HKU1, HCoV-NL63, and HCoV-OC43 spike in SARS-CoV-2 sero-negative populations (N = 887) by chronological age. SARS-CoV-2 sero-negative populations include all 2013 individuals (N = 407) and SARS-CoV-2 sero-negative individuals from 2020 (N = 480). Red dots indicate the geometric mean antibody titres for each HCoV among all individuals of that age (in years), with the black solid line connecting geometric means. Red bars represent upper and lower standard deviations. Black dashed lines describe the positivity cutoff for each antigen target.

Geometric mean IgG antibody titres for HCoV-229E, HKU1, NL63, and OC43 among SARS-CoV-2 negative individuals (N=887) from both 2013 (407/407; pre-pandemic) and 2020 (480/488; sero-negative for SARS-CoV-2) are shown in **Figure 1**. Initial seroconversion to all four HCoV was seen in all pediatric individuals ≤10 years of age, and sero-reactivity against HCoV was stable across age groups thereafter.

**Figure 2:**
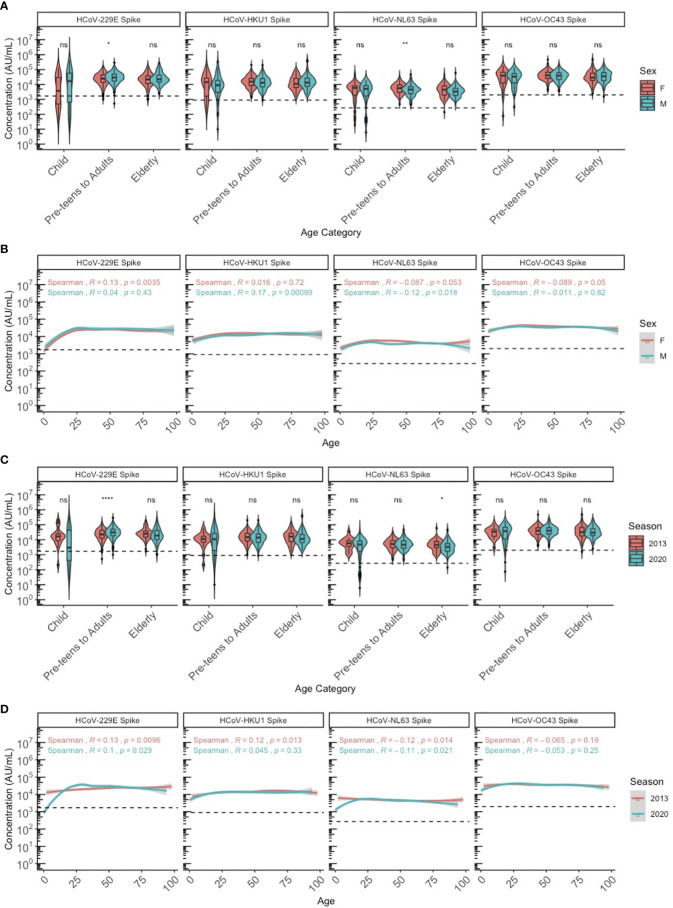
HCoV-229E, HCoV-HKU1, HCoV-NL63, and HCoV-OC43 spike IgG antibody levels between sexes and seasons (2013 and 2020) by chronological age among SARS-CoV-2 sero-negative persons (N = 887). **(A, B)** describe the HCoV antibody levels between male and female sexes by **(A)** age category and **(B)** chronological age. **(C, D)** describe the HCoV antibody levels between 2013 and 2020 seasons by **(C)** age category and **(D)** chronological age. In **(A, C)**, age categories are stratified according to children (≤10 years old), pre-teens to adults (11-69 years old), and the elderly (≥70 years old). Black dashed lines describe the positivity cutoff for each antigen target. Wilcoxon rank sum test was used to compare antibody levels between **(A)** sexes and **(C)** seasons. Spearman’s rank correlation was used to describe the relationship between antibody level and age in **(B, D)**, and Spearman’s correlation coefficient rho (R) and corresponding P-values (P) are reported. ns, not significant, * = p < 0.05, ** = p < 0.01 **** = p < 0.0001.

We stratified the population by three age categories to compare HCoV antibody levels between age groups, biological sex (male and female) and years as a proxy for different respiratory seasons (2013 and 2020): children (≤10 years old), pre-teens (adolescents) to adults (11-69 years old), and elderly adults (≥70 years old). Discrete categories for age groups were selected based on biological reasons and because we found that by 10 years of age, almost 100% of individuals had IgG to all 4 HCoV above the lower 95% confidence interval. HCoV-specific antibody levels were similar between males and females, although NL63 antibodies were significantly higher in female than male pre-teens/adults (P<0.05), and 229E antibodies were significantly higher in male than female pre-teens/adults (P<0.05) (**Figure 2A**). Males and females demonstrated very similar HCoV antibody trends across ages (**Figure 2B**), with no overall sex differences. Within the SARS-CoV-2 sero-negative population (N=887), antibody levels to HCoV showed minimal differences between the 2013 and 2020 seasons (**Figures 2C, D**). Pre-teens to adults in 2020 exhibited significantly higher levels of 229E antibodies when compared against the 2013 population (P<0.05), while the elderly in 2020 exhibited significantly lower levels of NL63 antibodies. Overall, no consistent differences in HCoV antibodies were observed between sampling year and sexes.

**Figure 3:**
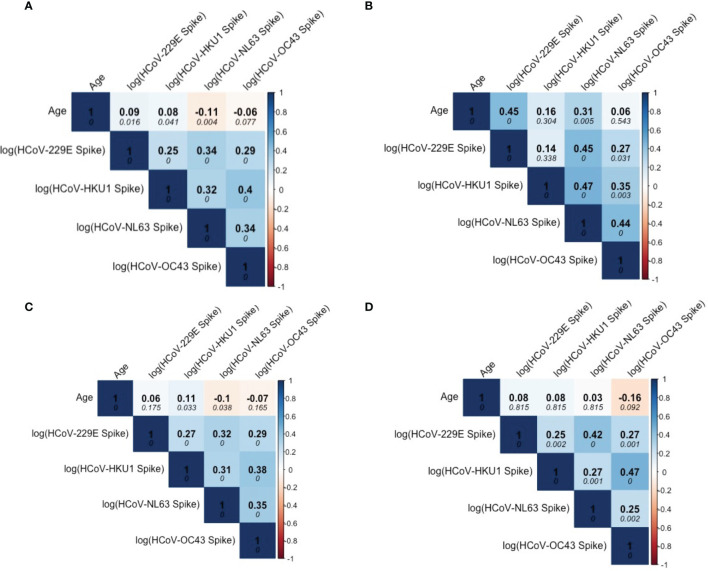
Correlation matrix describing relationship among Age and HCoV-specific IgG antibodies in SARS-CoV-2 sero-negative persons (N = 887). **(A)** describes correlations within the entire SARS-CoV-2 sero-negative population, comprised of all 2013 individuals (N = 407) and SARS-CoV-2 sero-negative individuals from 2020 (N = 480), which are further stratified by age categories: **(B)** children (≤10 years old) (N = 587), **(C)** pre-teens to adults (11-69 years old) (N = 104), and **(D)** the elderly (≥70 years old) (N = 196). Darker colours represent stronger correlations. Spearman’s correlation coefficient is reported in each matrix (top numerical value). P-values are reported in italics.

Among the SARS-CoV-2 sero-negative population (N=887), correlations were observed between age and HCoV antibody levels, specifically for HCoVs 229E and NL63 (**Figure 3A**). Overall, strong correlations between the four HCoV were seen in children (**Figure 3B**), pre-teens to adults (**Figure 3C**) and the elderly (**Figure 3D**). Additionally, age appears to be more strongly correlated with HCoV-229E and NL63 in children than in the other age categories.

### Antibodies Against SARS-CoV-1 and SARS-CoV-2, Stratified by Age, Sex, and Year

**Figure 4:**
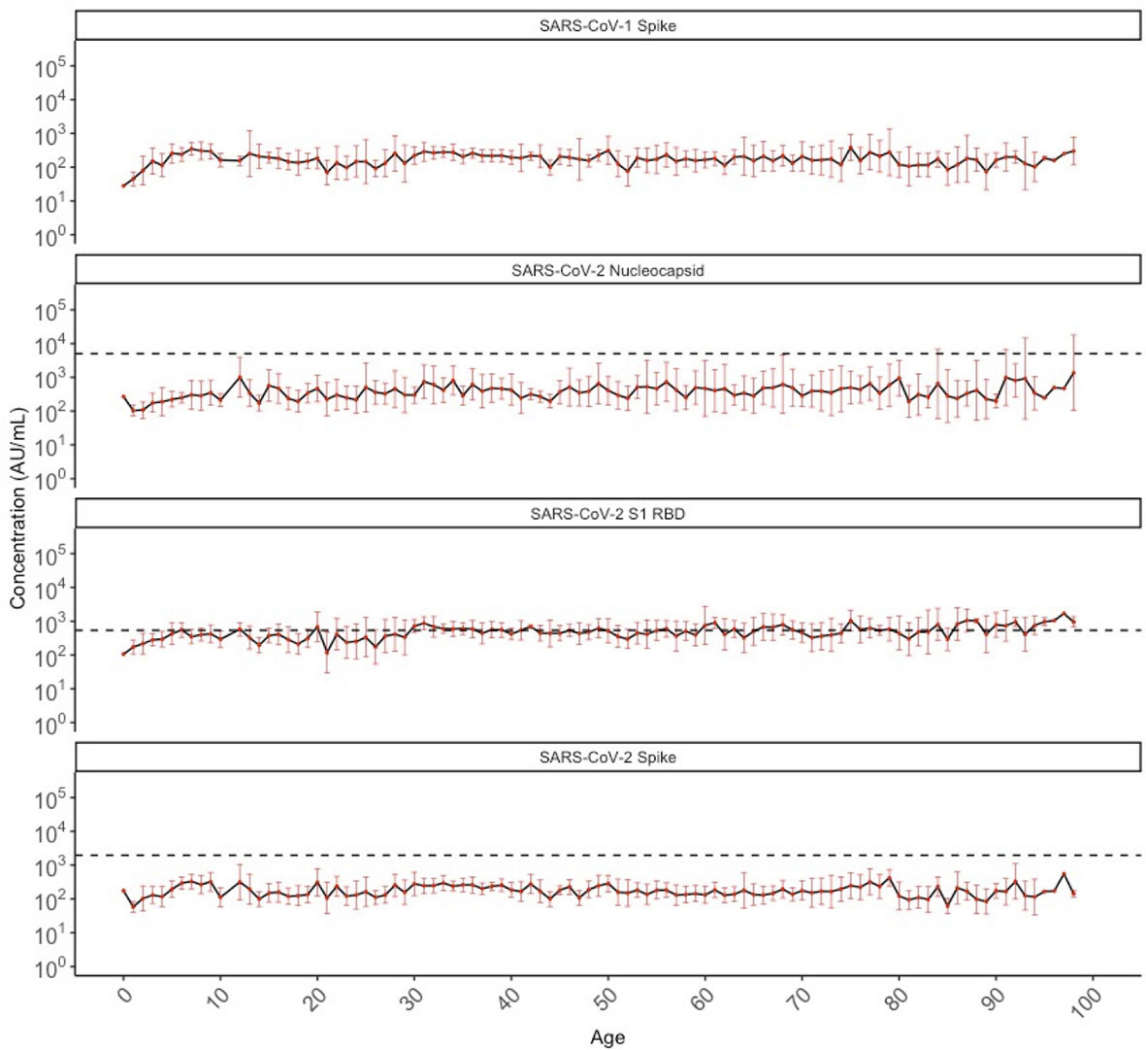
Geometric mean IgG antibody titres against SARS-CoV-1 spike and SARS-CoV-2 nucleocapsid, S1 RBD, and spike in SARS-CoV-2 sero-negative populations (N = 887) by chronological age. SARS-CoV-2 sero-negative populations comprise of all 2013 individuals (N = 407) and SARS-CoV-2 sero-negative individuals from 2020 (N = 480). Red dots indicate the geometric mean antibody titres for each HCoV among all individuals of that age (in years), with the black solid line connecting geometric means. Red bars represent upper and lower standard deviations. Black dashed lines describe the manufacturer provided positivity cutoff for each antigen target. No cutoff is available for SARS-CoV-1 spike.

We also evaluated the levels of cross-reactive IgG antibodies against SARS-CoV-1 and SARS-CoV-2 antigens in SARS-CoV-2 sero-negative persons. **Figure 4** shows the geometric mean antibody levels of target-specific SARS-CoV-2 cross-reactive antibodies among all sero-negative persons in our population (N=887), which are stable across chronological age after the age of 10 years. Anti-SARS-CoV-2 Nucleocapsid and Spike antibody levels were generally well below the manufacturer positivity cutoff; whereas, geometric mean SARS-CoV-2 RBD antibody levels meet or exceed the positivity cutoff across ages. While there are no validated cutoffs for SARS-CoV-1 positivity, geometric mean concentrations of cross-reactive antibodies appeared lower than those against SARS-CoV-2 and this was observed consistently across all ages.

**Figure 5:**
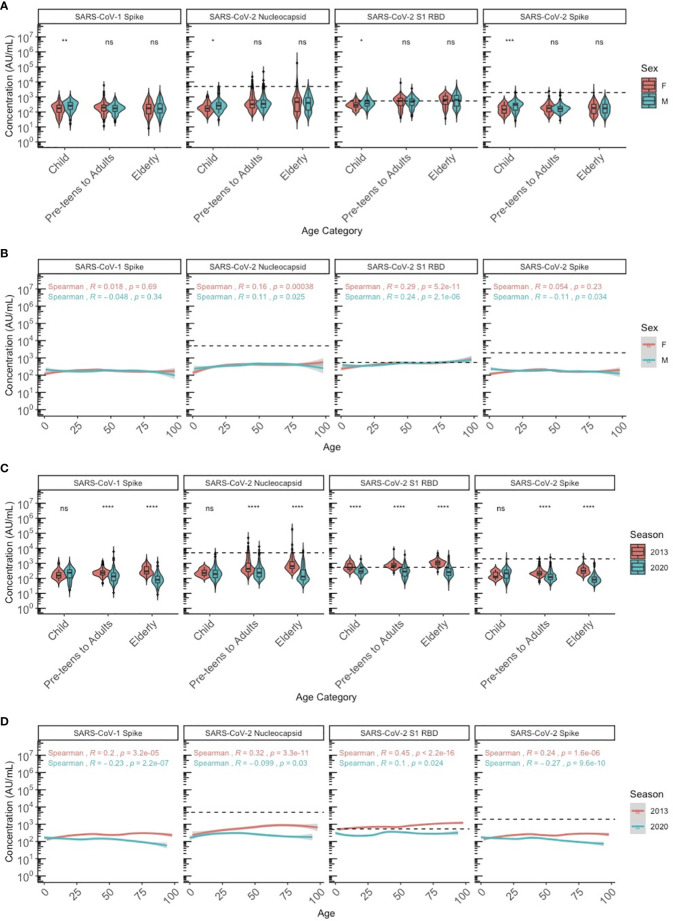
SARS-CoV-1 Spike and SARS-CoV-2 S1 RBD, nucleocapsid and spike IgG antibody levels between sexes and seasons by age among SARS-CoV-2 sero-negative persons (N=887). **(A, B)** describe the SARS-CoV-1 and SARS-CoV-2 cross-reactive antibody levels between male and female sexes by **(A)** age category and **(B)** chronological age. **(C, D)** describe the SARS-CoV-1 and SARS-CoV-2 cross-reactive antibody levels between 2013 and 2020 seasons by **(C)** age category and **(D)** chronological age. In **(A-C)**, age categories are stratified according to children (≤10 years old), pre-teens to adults (11-69 years old), and the elderly (≥70 years old). Black dashed lines describe the positivity cutoff for SARS-CoV-2 targets. Wilcoxon rank sum test was used to compare antibody levels between **(A)** sexes and **(C)** seasons. Spearman’s rank correlation was used to describe the relationship between antibody level and age in **(B, D)**, and Spearman’s correlation coefficient rho (R) and corresponding P-values (P) are reported. ns, not significant, * = p < 0.05, ** = p < 0.01, *** = p < 0.001 **** = p < 0.0001.

We also compared cross-reactive SARS-CoV-1 and SARS-CoV-2 antibodies between sexes across age categories among the SARS-CoV-2 sero-negative individuals (N=887). Male children had significantly higher cross-reactive antibodies against SARS-CoV-1 Spike and SARS-CoV-2 Spike, Nucleocapsid, and RBD compared to female children (**Figure 5A**). Antibody differences between sexes was not observed in the pre-teen to adult group and the elderly group for any of the SARS-CoV-1 and SARS-CoV-2 targets. Notably, cross-reactive SARS-CoV-2 RBD antibodies do appear to increase slightly with age for both males (Spearman’s rho = 0.24) and females (Spearman’s rho = 0.29), with older ages exceeding the positivity cutoff (**Figure 5B**).

Cross reactive antibodies against SARS-CoV-1 and SARS-CoV-2 targets were similar among children in both 2013 and 2020, except for SARS-CoV-2 RBD, for which pediatric individuals in 2013 showed significantly higher levels of cross-reactivity that mostly exceeded the positivity cutoff, compared to 2020 (**Figures 5C, D**). Among adults and the elderly, cross-reactive antibody levels against SARS-CoV-1 spike and SARS-CoV-2 Spike, Nucleocapsid, and RBD in 2013 were each significantly higher than in 2020. However, with the exception of SARS-CoV-2 RBD, most remained below the positivity cutoff.

Sera from 2013 and 2020 were dichotomized as negative or positive for antibodies against SARS-CoV-2 RBD, Nucleocapsid, and Spike in **Table 2**. Notably, 4.9% (20/407) of the pre-pandemic individuals from 2013 were deemed SARS-CoV-2 positive based on exceeding cut-off values for at least two of three MSD targets. When evaluating antibodies specific to each SARS-CoV-2 target separately, 2013 pre-pandemic individuals exhibited antibodies above the positivity cutoff for Nucleocapsid (4.7%, 19/407), RBD (81%, 329/407), and Spike (0.5%, 2/407).

**Table 2:** Population above positivity cut-off for SARS-CoV-2 targets in 2013 and 2020.

		2013 (N=407)	2020 (N=488)		CLIA+ (N=40)
	MSD Status	Overall	Negatives	Positives	Overall
**Overall SARS-CoV-2 Sero-status**	**Negative**	387 (95%)	480 (98.4%)	0	21 (52%)
	**Positive**	20 (4.9%)	0	8 (1.6%)	19 (48%)
**Nucleocapsid**	**Negative**	388 (95%)	475 (99%)	0 (0%)	21 (52%)
	**Positive**	19 (4.7%)	5 (1.0%)	8 (100%)	19 (48%)
**RBD**	**Negative**	78 (19%)	397 (83%)	0 (0%)	14 (35%)
	**Positive**	329 (81%)	83 (17%)	8 (100%)	26 (65%)
**Spike**	**Negative**	405 (99.5%)	478 (99.6%)	7 (88%)	24 (60%)
	**Positive**	2 (0.5%)	2 (0.4%)	1 (12%)	16 (40%)

CLIA+ = 40 samples positive for at least one SARS CoV-2 target on chemiluminescent assays supplemented.

Among the 2020 population, 1.6% (8/488) were deemed SARS-CoV-2 sero-positive based on exceeding cut-off values for at least two of three targets as per the validated MSD algorithm. The sera from these individuals were collected in May 2020 survey, and 1/8 were children, 4/8 were pre-teens to adults, and 3/8 were from the elderly group. All 8 individuals were seropositive for Nucleocapsid and RBD, while 7/8 individuals were also sero-positive for Spike. Among the remaining 98.4% (480/488) individuals from 2020 who were sero-negative for SARS-CoV-2 by MSD testing, some individuals also exhibited cross-reactive antibodies above the cutoff for Nucleocapsid (1%, 5/480), RBD (17%, 83/480), and Spike (0.4%, 2/480). While the percent with cross-reactive antibodies against SARS-CoV-2 Nucleocapsid and Spike was similar between the 2013 and 2020 population, this differed for RBD (**Table 2** and **Figure 5D**).

Among the series of 40 samples across serial sero-surveys in March, May and September 2020 that were positive for at least one SARS-CoV-2 target on commercial CLIA, 19 were SARS-CoV-2 sero-positive according to the MSD algorithm requiring dual positivity. These 19 individuals were sourced from the March (1/19), May (4/19), and September (14/19) 2020 surveys and comprised of 1/19 children, 16/19 preteens to adults, and 2/19 elderly. Together with the 8/488 considered SARS-CoV-2 sero-positive described above, there were a total of 27/935 (3%) individuals considered SARS-CoV-sero-2 positive in this study (two children, 20 pre-teens to adults, 5 elderly).

### Association of HCoV Antibodies and SARS-CoV-2 Cross Reactive Antibodies

**Figure 6:**
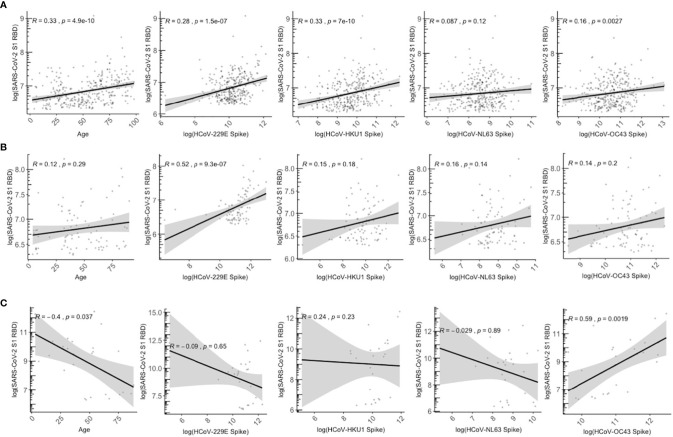
Relationship between age and HCoV-specific IgG antibodies with SARS-CoV-2 S1 RBD antibodies among SARS-CoV-2 overall sero-negative persons who are positive for SARS-CoV-2 S1 RBD antibodies. **(A)** describes all individuals from 2013 season who have SARS-CoV-2 S1 RBD antibodies above positivity cutoff (N = 329). **(B)** describes SARS-CoV-2 sero-negative individuals from 2020 who have SARS-CoV-2 S1 RBD above positivity cutoff (N = 87). **(C)** describes SARS-CoV-2 sero-positive individuals from 2020 who also have SARS-CoV-2 S1 RBD above positivity cutoff (N = 27). SARS-CoV-2 sero-positive individuals are from the serosurvey cohort (N = 8) and the known positive controls added (N = 27). Gray shaded area indicates 95% confidence intervals. Spearman correlations were used to describe the relationship between samples, and corresponding Spearman correlation coefficient (R) and p-values are reported.

We assessed the association between age and HCoV antibodies with detection of SARS-CoV-2 cross-reactive antibodies exceeding cutoff values in presumably unexposed persons. Specifically, among those with SARS-CoV-2 RBD antibodies above the positivity cutoff, we evaluated the correlation between age and HCoV antibody levels vs. SARS-CoV-2 cross-reactive RBD antibody levels among the 2013 population collected pre-COVID-19 pandemic (N=329), 2020 SARS-CoV-2 sero-negatives (N=83), and SARS-CoV-2 sero-positive individuals (N=27) (**Figure 6**). Among the SARS-CoV-2 sero-negative individuals, age and HCoV antibody titres show a weakly positive relationship with SARS-CoV-2 crossreactive RBD antibodies, such that older age and higher HCoV titres are correlated with the presence of more cross-reactive SARS-CoV-2 RBD antibodies (**Figures 6A, B**). Among the four HCoVs, 229E and HKU1 exhibit the strongest correlations (**Figures 6A, B**).

In contrast, the positive association is lost in SARS-CoV-2 sero-positive individuals (**Figure 6C**), who demonstrate a weakly negative trend between SARS-CoV-2 RBD antibodies with HCoVs 229E, NL63, and HKU1, although associations were not significant and large 95% confidence intervals were observed. Lower levels of anti-SARS-CoV-2 RBD antibodies were associated with older age (R=-0.4, P=0.037). Higher OC43 titres were positively correlated with higher anti-SARS-CoV-2 RBD antibodies (R = 0.59, P=0.0019).

Multivariable linear regression was also conducted to identify whether age, sex, and HCoV antibody levels independently predict the magnitude of SARS-CoV-2 cross-reactive RBD antibodies (**Table 3**). Age, HCoV-229E titres, and HCoV-HKU1 titres were significantly associated (P<0.05) with cross-reactive RBD antibodies for the 2013 SARS-CoV-2 negative population (N=329), although only HCoV-229E remained a significant predictor of SARS-CoV-2 RBD antibody levels in the SARS-CoV-2 sero-negative population in 2020 (N=83). Among SARS-CoV-2 sero-positive individuals (N=27), only OC43 was independently associated with SARS-CoV-2 RBD antibody titres (P<0.05).

**Table 3:** Multiple linear regression analysis of anti-SARS-CoV-2 RBD IgG antibody levels above positivity cutoff in SARS-CoV-2 sero-negative individuals (2013 and 2020) and SARS-CoV-2 sero-positive individuals, with adjustment for age, sex, and anti-HCoV IgG signals.

Variable	2013 (N = 329)		2020 (N = 83)		SARS-CoV-2 Sero-Positives (N = 27)	
	Coefficient	P-value	Coefficient	P-value	Coefficient	P-value
(Intercept)	4.009	–	3.685	–	-7.787	–
Age	0.005	<0.001	0.001	0.461	-0.029	0.127
Sex (Male)	0.035	0.372	0.021	0.818	-0.606	0.380
log(HCoV-229E Spike)	0.116	<0.001	0.0215	<0.001	0.734	0.175
log(HCoV-HKU1 Spike)	0.087	<0.001	0.065	0.183	-0.251	0.648
log(HCoV-NL63 Spike)	0.022	0.379	0.047	0.449	-0.549	0.252
log(HCoV-OC43 Spike)	0.031	0.243	-0.036	0.629	1.604	0.023
Adjusted R-squared	0.233		0.195		0.335	

In contrast, age and HCoV antibody level did not independently predict cross-reactive Nucleocapsid titers. With only two individuals from 2013 exhibiting SARS-CoV-2 spike antibody levels above the positivity cutoff (**Table 2**), we could not conduct these analyses for that target.

### HCoV Antibodies in SARS-CoV-2 Positive Individuals

**Figure 7:**
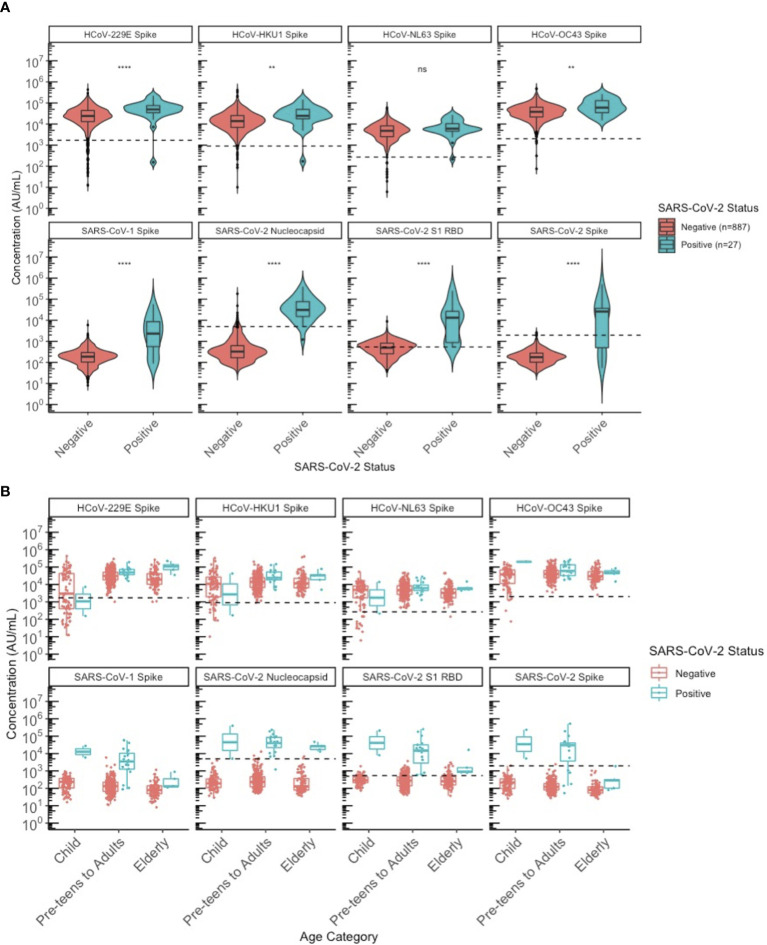
Comparison of HCoV-specific and SARS-CoV-2 specific IgG antibody levels between SARS-CoV-2 sero-negative and sero-positive individuals. **(A)** describes the entire population from 2013 and 2020. All 2013 individuals were classified as sero-negative for SARS-CoV-2 status, regardless of whether they appeared SARS-CoV-2 sero-positive by MSD algorithm or not. **(B)** describes only the 2020 population stratified by age category: children (≤10 years old), pre-teens to adults (11-69 years old), and the elderly (≥70 years old). SARS-CoV-2 sero-positive individuals include children (N = 2), pre-teens to adults (N = 20), elderly (N = 5); similar to SARS-CoV-2 sero-negative individuals where the distribution by age category is children (N = 88), pre-teens to adults (N = 295), elderly (N = 97). Black dashed lines describe the positivity cutoff for each antigen target. ns, not significant, ** = p < 0.01, **** = p < 0.0001.

Finally, we wanted to evaluate how HCoV antibody levels differ between SARS-CoV-2 sero-negative (N=887) and sero-positive (N=27) individuals. The 27 sero-positive individuals used for this analysis included the 8/488 from the 2020 cohort together with the 19 supplemental SARS-CoV-2 known positives. In addition to their higher mean SARS-CoV-2 antibody levels, these SARS-CoV-2 sero-positive individuals also had higher SARS-CoV-1, HCoV-229E, HKU1, and OC43 antibody levels (**Figure 7**).

We attempted to stratify this by age categories to evaluate differences between the SARS-CoV-2 sero-positive and sero-negative populations across ages (**Figure 7**). The small sample size of sero-positive specimens overall precluded statistical comparisons by age group but the observation that SARS-CoV-2 sero-positive individuals had higher SARS-CoV-1 and HCoV antibodies did not appear to be limited to or driven by one age category but was generally seen for all age groups.

## Discussion

In this study we explored age-based seroprevalence to the endemic HCoVs, HCoV-229E, HCoV-NL63, HCoV-HKU1, and HCoV-OC43 in BC, Canada. Retrospective sampling of anonymized residual sera obtained from an outpatient laboratory from 2013 (pre-COVID-19 pandemic) and early 2020 (first wave of the pandemic) was used to interrogate the association between HCoV and SARS-CoV-2 cross-reactive antibodies in presumably unexposed individuals. By using a high-throughput pan-coronavirus multiplex immunoassay with age-stratified sampling, we reinforce earlier findings that priming exposures to HCoVs occur during early childhood and remain stable into older age (9, 20, 23). Furthermore, we demonstrate HCoV antibodies are associated with cross-reactive antibodies against SARS-CoV-2, notably the anti-RBD IgG.

No overall differences in population HCoV antibody levels were observed between respiratory seasons (2013 and 2020 collection years), except for 229E among adults and NL63 among the elderly. No overall sex differences were observed in HCoV antibody levels, apart from small differences among adults for 229E and NL63. Given that circulating HCoV strains may differ each season (23), and different age groups may be subjected to varying exposures, slight differences in antibody levels between seasons and sexes are expected. Nonetheless, no overall sex and seasonal differences in HCoV antibody levels across ages were consistently observed in our study population.

In addition to SARS-CoV-2 RBD, we identified cross-reactive antibodies, albeit to a lesser extent, against SARS-CoV-2 Spike and Nucleocapsid and SARS-CoV-1 Spike in 2013 and 2020, consistent with other published findings (19, 24). The greater cross-reactivity observed in anti-RBD in comparison to anti-spike is not unexpected due to the larger % CVs and 95% confidence intervals for specificity of RBD, as we previously described in our assay validations (22). Additionally, the MSD assay utilizes recombinant proteins printed onto the surface of the plate, and does not account for any post-translational modifications occurring *in vivo*. Recent *in vitro* studies have demonstrated the importance of post-translational modifications, such as glycosylation, on the immunogenicity of RBD (25). As such, cross-reactivity observed on the MSD assay may not be fully representative of biological cross-reactivity. However, we believe our findings still capture the biological interactions, due to the large differentials observed between positive and negative antibodies in our assay validations.

Interestingly, we found male children demonstrated higher levels of cross-reactive antibodies. While sex has been identified as an independent prognostic factor for COVID-19 (26), very few studies, if any, have interrogated sex differences in SARS-CoV-2 cross-reactive antibodies. However, the absence of any consistent sex differences among adults in our cohorts suggests that the increased severity observed among male COVID-19 patients is unlikely to be due to pre-existing cross-reactive SARS-CoV-2.

Notably, SARS-CoV-2 cross-reactive antibodies were consistently higher among the population in 2013 than in 2020 across all ages (**Figures 4C, D**), with a substantial proportion of the 2013 cohort having anti-RBD antibodies above the positivity cutoff. While it may be suspected that this is due to inter-run variability, our assay is well-validated and any observed cross-reactivity for RBD were unlikely due to assay inter-run factors, but rather a true phenomenon for the currently defined cutoffs. It is also important to note that the 2020 cohort was sampled during the first wave of the COVID-19 pandemic in BC, when public health measures successfully suppressed SARS-CoV-2 circulation as well as the transmission of other respiratory viruses. In this case, the 2020 population in this study may have had less exposure to other respiratory viral pathogens, including HCoVs. Further studies investigating the comprehensive contributions of respiratory viral pathogens may help explain the little difference observed in HCoV antibodies, but substantial difference in cross-reactive anti-RBD antibodies, observed between the 2013 and 2020 seasons.

Here, we show that in the presumed absence of SARS-CoV-2 exposure, age, HCoV-229E, and HKU1 antibodies, are positively correlated and significant predictors of SARS-CoV-2 cross-reactive RBD antibodies. However, the low R-squared values indicate that other unexplained factors contribute to the observed variation. Our findings add to the very few other studies that have assessed the association between alpha-CoVs and cross-reactive SARS-CoV-2 antibodies, similarly suggesting an association between HCoV-229E and cross-reactive anti-RBD IgG antibodies in unexposed individuals (9, 19).

The source of cross-reactive antibodies to SARS-CoV-2 has not been extensively studied, and the extent to which they may influence the severity of COVID-19 or long COVID remains unknown. Evidence indicates that a recent HCoV infection is associated with less severe disease, including lower intensive care unit (ICU) admission rates and a higher probability of survival (27, 28), suggesting that HCoV responses may protect against COVID-19 progression. In contrast, other studies have demonstrated that the cross-reactive antibodies do not protect against COVID-19 or neutralize SARS-CoV-2 (29, 30), suggesting a potential role for “original antigenic sin” (OAS) cannot be ruled out. In OAS, immune response to a new or evolved virus is biased by past exposures to a closely related pathogen. OAS may preferentially boost memory responses from the initial exposure, at the expense of generating new immune responses against epitopes specific to the current infection. Our findings neither confirm nor refute a role for OAS but provide further understanding of possible immunological interactions or cross-reactivity that could underpin protective or untoward effects and on that critical basis warrant further investigation.

There are several limitations to this study. As a cross-sectional sero-survey, we were only able to describe associations between HCoV and SARS-CoV-2 cross-reactive antibodies. To further understand causation, future studies utilizing longitudinal cohorts would be highly beneficial. Delays in generating memory antibody responses and timing of the blood draws could also affect the detection of CoV specific antibodies, which may lead to an overall underestimation of seroprevalence (6, 21, 31). In addition, our study did not assess the potential protective effects of these antibody responses. For example, although RBD-binding antibodies are strongly correlated with neutralizing activity (32), gold standard neutralization assays might provide additional insights into the functional importance of SARS-CoV-2 cross-reactive antibodies between age groups. Our study also includes a small SARS-CoV-2 sero-positive population, especially when stratified by age categories, precluding our ability to conduct age-stratified comparisons between SARS-CoV-2 sero-positive versus sero-negative individuals.

In summary, our data reinforces that initial exposure and seroconversion to endemic HCoVs occurs during early childhood and that SARS-CoV-2 cross-reactive antibodies are detected among unexposed populations. Anti-HCoV antibodies appear to be associated with SARS-CoV-2 cross-reactive antibodies. Specifically, we found that HCoV-229E (and HCoV-HKU1 for 2013) antibody titres appeared to be positively associated with cross-reactive RBD antibodies for both the 2013 and 2020 SARS-CoV-2 negative populations. However, we are only able to draw associations, but not infer any causation, as our study was a cross-sectional study. Additionally, our results also demonstrate that there are likely other unexplained factors that can influence SARS-CoV-2 cross-reactive antibodies. The finding of SARS-CoV-2 cross-reactive antibodies, particularly to RBD in a large proportion of unexposed individuals, also highlights the importance of utilizing multiple targets when diagnosing SARS-CoV-2 seropositivity to improve the positive predictive value of serological diagnosis. Further investigation into the durability and functionality of the antibody response will clarify the role of cross-reactive antibodies for natural and vaccine-induced protection against SARS-CoV-2. These investigations are particularly critical now in the context of more relaxed public health measures related to SARS-CoV-2 pandemic control, particularly with the subsequent re-emergence of various respiratory viruses including HCoVs, as they may shed light on the relationship between HCoV exposures and COVID-19 disease severity and duration.

## Data Availability Statement

The raw data supporting the conclusions of this article will be made available by the authors, without undue reservation.

## Ethics Statement

The studies involving human participants were reviewed and approved by Clinical Research Ethics Board of the University of British Columbia (H20-00653). Written informed consent from the participants’ legal guardian/next of kin was not required to participate in this study in accordance with the national legislation and the institutional requirements.

## Author Contributions

AJ, DS, SG, and MK conceptualized the study. AJ, DS, SG, and IS were awarded funding for the project (PI AJ). DS, DP, and RR selected and contributed samples for testing. AJ, DS, SG, IS, AL, GT, and SK were involved in the overall study design. GT, AL, SK, IS, MM, AJ, and MI were involved in performing laboratory methods and analysis of results. GT and AL drafted the manuscript. All authors reviewed and edited the manuscript. AJ, IS, MK, SG, and DS critically advised on the reported conclusions. All authors contributed to the article and approved the submitted version.

## Funding

This work was supported by the Michael Smith Foundation for Health Research [Grant number: COV-2020–1120].

## Conflict of Interest

Author RR was employed by company LifeLabs.

The remaining authors declare that the research was conducted in the absence of any commercial or financial relationships that could be construed as a potential conflict of interest.

## Publisher’s Note

All claims expressed in this article are solely those of the authors and do not necessarily represent those of their affiliated organizations, or those of the publisher, the editors and the reviewers. Any product that may be evaluated in this article, or claim that may be made by its manufacturer, is not guaranteed or endorsed by the publisher.
